# Interrelationships between BMI, skinfold thicknesses, percent body fat, and cardiovascular disease risk factors among U.S. children and adolescents

**DOI:** 10.1186/s12887-015-0493-6

**Published:** 2015-11-18

**Authors:** David S. Freedman, Cynthia L. Ogden, Brian K. Kit

**Affiliations:** Division of Nutrition, Physical Activity, and Obesity, Centers for Disease Control and Prevention, Atlanta, GA USA; National Center for Health Statistics, Centers for Disease Control and Prevention, Hyattsville, MD USA

**Keywords:** BMI, Skinfold thicknesses, Body fat, DXA, Children, NHANES

## Abstract

**Background:**

Although the estimation of body fatness by Slaughter skinfold thickness equations (PBF_Slaughter_) has been widely used, the accuracy of this method is uncertain. We have previously examined the interrelationships among the body mass index (BMI), PBF_Slaughter,_ percent body fat from dual energy X-ray absorptiometry (PBF_DXA_) and CVD risk factor levels among children who were examined in the Bogalusa Heart Study and in the Pediatric Rosetta Body Composition Project. The current analyses examine these associations among 7599 8- to 19-year-olds who participated in the (U.S.) National Health and Nutrition Examination Survey from 1999 to 2004.

**Methods:**

We analyzed (1) the agreement between (1) estimates of percent body fat calculated from the Slaughter skinfold thickness equations and from DXA, and (2) the relation of lipid, lipoprotein, and blood pressure levels to BMI, PBF_Slaughter_ and PBF_DXA_.

**Results:**

PBF_Slaughter_ was highly correlated (r ~ 0.85) with PBF_DXA_. However, among children with a relatively low skinfold thicknesses sum (triceps + subscapular), PBF_Slaughter_ underestimated PBF_DXA_ by 8 to 9 percentage points. In contrast, PBF_Slaughter_ overestimated PBF_DXA_ by 10 points among boys with a skinfold thickness sum ≥ 50 mm. After adjustment for sex and age, lipid levels were related similarly to the body mass index, PBF_DXA_ and PBF_Slaughter_. There were, however, small differences in associations with blood pressure levels: systolic blood pressure was more strongly associated with body mass index, but diastolic blood pressure was more strongly associated with percent body fat.

**Conclusions:**

The Slaughter equations yield biased estimates of body fatness. In general, lipid and blood pressure levels are related similarly to levels of BMI (following adjustment for sex and age), PBF_Slaughter,_ and PBF_DXA_.

## Background

The body mass index (BMI, kg/m^2^) is widely used as a screening tool to identify obese children, and a high BMI in early life is associated with adverse levels of cardiovascular disease risk factors and the initial stages of atherosclerosis [1]. Although children and adolescents with a high BMI level also tend to have a high level of body fatness [2], BMI is composed of both fat mass and lean body mass, and it can be a poor indicator of fatness among those who have normal or relatively low levels of percent body fat [3, 4].

Despite the large measurement errors associated with skinfold thicknesses [5, 6], skinfold thicknesses are widely used among children and adolescents [7–9] to assess body fatness. Although several investigators have found the levels of percent body fat estimated from skinfold thickness equations [3, 10, 11] are more strongly correlated with more accurate estimates of body fatness than is BMI, this does not necessarily mean that skinfolds are better predictors of adverse levels of cardiovascular disease (CVD) risk factors. Several studies of children and adults have found that BMI is as strongly associated with levels of lipids, blood pressure and insulin as are more accurate estimates of body fatness [12–20]. This similarity may result from the independent association of lean body mass to adverse levels of several CVD risk factors [15] or from the errors associated with either skinfold thickness measurements [5] or the equations that are used estimate body fatness [21].

We have previously reported that BMI and skinfold thicknesses were related similarly to levels of CVD risk factor levels among children and adolescents who in the Bogalusa Heart Study [19]. The objectives of the current study were to (1) assess the accuracy of the Slaughter skinfold thickness equations in the estimation of percent body fat (PBF_Slaughter_) for levels of percent body fat calculated form dual energy X-ray absorptiometry (PBF_DXA_), and (2) compare the magnitudes of the relations of levels of CVD risk factors to levels of PBF_DXA_, PBF_Slaughter_, and BMI levels among children and adolescents. These associations are examined among these 7599 8- to 19-year-olds who participated in the U.S. National Health and Nutrition Examination Survey (NHANES), 1999–2004.

## Methods

### Ethics statement

The procedures for NHANES were in accord with the ethical standards of CDC, and the protocols were approved by the National Center for Health Statistics Research Ethics Review Board. No approval was required for the current analyses, and the data are publicly available at http://www.cdc.gov/nchs/nhanes/nhanes_questionnaires.htm.

### Study population

The 1999–2004 NHANES is a representative, cross-sectional sample of the U.S. civilian, non-institutionalized population. Parental permission was obtained for minors under the age of 18 years; 7- to 17-year-olds also provided documented assent. Consent was obtained for all adults, 18 years and older. Race and ethnicity were self-reported, and we classify subjects as non-Hispanic white, non-Hispanic black, Mexican American and other. The overall examination response rate for 6- to 19-year-olds in NHANES 1999–2004 was 85 % [22]. The current analyses included 7599 8- to 19-year-olds (see below).

### DXA examinations

DXA scans were acquired in NHANES 1999–2004 for boys and non-pregnant girls who were at least 8 years of age using a Hologic QDR 4500A fan-beam densitometer (Hologic Inc., Bedford MA) [23, 24]. Scans were analyzed using Hologic Discovery software (version 12.1). Percentage body fat from DXA (PBF_DXA_) was calculated as 100 × (DXA estimated total fat mass ÷ DXA estimated total mass).

We used the NHANES DXA Multiple Imputation Data Files [24] in the analyses. About 10 % of the children and adolescents in the current study were missing at least one DXA measurement, and because missingness was related to BMI and other characteristics, an analysis restricted to the non-missing values could be biased. The 1999–2000 DXA data for 8- to 17-year-old girls are available only in the Research Data Center, and these data are not used in the current analyses. We do, however, use the 1999–2000 data from 18- and 19-year-old girls. There were 7599 children and adolescents who had data for both PBF_DXA_ (either calculated or imputed) and BMI in the current study.

### BMI and skinfold thicknesses

Body weight and height were measured using standardized techniques, and BMI (kg/m^**2**^) was calculated as a measure of relative weight. BMI-for-age z-scores (SDs) and percentiles were calculated for each child based on the CDC Growth Charts [25]; these values express the BMIs of the examined 8- to 19-year-olds relative to their sex-age peers in the U.S. between 1963 and 1980. A child with a BMI-for-age ≥ 95^th^ percentile of the CDC reference population is considered to be obese, and 120 % of the 95^th^ percentile [26] is used as the cutoff for extreme obesity.

Because BMI z-scores based on the CDC growth charts have several limitations, including an upper limit of about 3.0 at most ages [27], several analyses are based on the residuals of regression models in which BMI was predicted by age (modeled using restricted cubic splines) within each sex. These residuals represent a child’s BMI relative to other children of the same sex and age in the current study in kg/m^**2**^ units (rather than as SD scores), and we refer to these values as ‘adjusted BMI’. It has been shown [28] that BMI is preferable to BMI-for-age z-scores when examining longitudinal changes.

The thickness of the triceps and subscapular skinfolds were measured to the nearest 0.1 mm using Holtain skinfold calipers. These data were missing for about 7 % (subscapular) and 4 % (triceps) of children in the current study because of measurement difficulties. We used the *Amelia II* package in R [29, 30] to impute missing skinfold thicknesses from sex, race, age, BMI, PBF_DXA_, and CVD risk factors. We used the logarithm of the skinfold thickness in the imputations to improve normality.

We estimated PBF_Slaughter_ from equations in Slaughter et al. [31]. This set of equations incorporates linear and squared terms for the sum of the thicknesses of the subscapular and triceps skinfolds (SF sum), along with sex, maturation, and race (white/black) to estimate percent body fat. The intercepts and slopes of these equations differ by sex and SF sum; they also differ by maturation stage and race among boys who have a SF sum < 35 mm. As has been done in other investigations [7], we used the age of the child as a surrogate for sexual maturation: boys <12 y were considered pre-pubescent, those 12.0 to 13.9 y as pubescent, and those ≥ 14 y as post-pubescent. The equations for white boys were used to estimate percent body fat among all non-black boys.

### Lipids and blood pressure

Serum levels of lipids and high-density lipoprotein (HDL) cholesterol were measured for NHANES participants aged ≥ 3 y [32, 33]. Fasting levels of triglycerides (TG) were available for participants aged ≥ 12 y who reported that they had fasted for 8.5 – 23 h before the morning examination [32]. For fasting TG levels <400 mg/dL, low-density-lipoprotein (LDL) cholesterol was calculated from the Friedewald equation [34]. Levels of TG were skewed and were log-transformed in all analyses.

Blood pressure measurements were taken in the mobile examination center after the participants rested quietly in a sitting position for 5 min. Three consecutive blood pressure readings were attempted, and if a measurement was interrupted or incomplete, a fourth attempt was made. The mean of these determinations was used to calculate blood pressure z-scores and percentiles relative to a child’s sex, age and height [35].

Of the 7599 subjects who had data on BMI and PBF_DXA_, 735 did not have a lipid measurement and 245 did not have a SBP or DBP. These subjects, along with an additional 153 children who reported being told that they had diabetes or were taking drugs that affect lipid or blood pressure levels, were excluded from the risk factor analyses. These exclusions resulted in the samples for the analyses of CVD risk factors consisting of 7311 (SBP and DBP), 6735 (TC), and 6733 (HDLC) subjects. Sample sizes for the analyses of fasting levels of TG and LDL-C were 2301 and 2291, respectively.

### Statistical analyses

Analyses were performed using the *survey* and *mitools* packages in R [30, 36], and all analyses account for the sample weights, sample design and multiple imputations. NCHS provided 5 complete DXA Multiple Imputation Data Files [24], in which the missing DXA estimates were imputed using multiple imputation [37]. For the missing skinfold thickness data, we imputed 1 estimate in each of these 5 DXA datasets using information on sex, age, BMI, DXA measurements, non-missing skinfold values, sample weights and other characteristics; this yielded 5 datasets that had complete information for both the DXA and skinfold thickness measurements. We accounted for the uncertainty of the imputed values by analyzing each of the 5 datasets separately and then combining the results [38–41].

The agreement between levels of PBF_DXA_ and PBF_Slaughter_ was assessed in Bland-Altman plots [42], in which the mean of the 2 estimates of percent body fat (x-axis) is plotted vs. the difference (y-axis: PBF_Slaughter_ - PBF_DXA_). We also examined levels of PBF_DXA_ and PBF_Slaughter_ by sex and levels of the SF sum; 4 categories the SF sum (approximately the sex-specific 33^rd^, 67^th^ and 90^th^ percentiles) were used in these analyses. We used lowess which accounted for the sample weights, to graphically examine the relation of SF sum to levels of PBF_DXA_ and PBF_Slaughter_. The y-axis of the lowess curves represents the mean of the estimated values over the 5 imputations.

We then examined the weighted correlations between BMI, PBF_Slaughter_ and PBF_DXA_ with levels of the CVD risk factors. To control for the influence of age, these analyses used the residuals from sex-specific regression models in which each characteristic was regressed on age. The statistical significance of the observed differences (e.g., are levels of HDL cholesterol more strongly correlated with PBF_DXA_ than with adjusted BMI?) were based on jackknife replicate weights which were calculated using the ‘withReplicates’ function of the *survey* package [36]. Variances were then combined across the imputations.

## Results

Various characteristics of the sample are shown among boys and girls in Table 1. About 18 % of the children were obese, with 6 % considered to be extremely obese (BMI ≥ 120 % of the CDC 95^th^ percentile). Mean levels of the SF sum, PBF_DXA_ and PBF_Slaughter_ were about 30 to 40 % higher among girls than boys (*p* < 0.001 for all comparisons). As seen in the final 2 rows of Table 1, the Slaughter estimate of percent body fat, however, substantially underestimated the mean PBF_DXA_ among both boys (by 4 percentage points) and girls (by 6 percentage points); *p* < 0.001 for both comparisons). Additional sex-specific analyses indicated that PBF_DXA_ was more strongly correlated with both PBF_Slaughter_ and the SF sum (*r* = 0.82 to 0.86) than with BMI-for-age (*r* = 0.75 to 0.80). Whereas mean levels of PBF_DXA_ generally increased with age among girls, mean levels decreased among boys between the ages of 12 and 16 y (data not shown).

**Table 1:** Descriptive Characteristics of the Sample ^a^

Characteristic	Boys (*n* = 4493)	Girls (*n* = 3106)
Race/Ethnicity		
Non-Hispanic White	61 %	62 %
Non-Hispanic Black	15 %	15 %
Mexican-American	11 %	11 %
Other	7 %	7 %
Age (y)	13.9 ± 0.1	13.9 ± 0.1
BMI (kg/m^2^)	21.8 ± 0.1	22.2 ± 0.2
BMI-for-age (z-score) ^b^	0.46 ± 0.03	0.51 ± 0.04
Obese (%) ^c^	18 ± 1	17 ± 1
Extreme Obesity (%) ^c^	6 ± 1	6 ± 1
Subscapular skinfold thickness (mm)	9.1 ± 0.2	12.8 ± 0.3
Triceps skinfold thickness (mm)	11.2 ± 0.2	17.4 ± 0.3
Skinfold thickness sum (mm)	20.2 ± 0.5	30.8 ± 0.6
Slaughter estimated body fat (%)	21.1 ± 0.3	27.4 ± 0.3
DXA calculated body fat (%)	25.4 ± 0.2	33.3 ± 0.3

^a^Values represent prevalences or means (± SE). Because the skinfold thickness measures were skewed, values for these 3 variables represent estimates of the medians and their SEs

^b^Z-score (standard deviation score) of children relative to the 2000 CDC growth charts

^c^Obesity is defined as a BMI-for-age ≥ 95th percentile of the CDC reference population or a BMI ≥ 30 kg/m^2^. Extreme obesity is defined as a BMI-for-age ≥ 120 % of the 95th percentile [26]

**Fig. 1:**
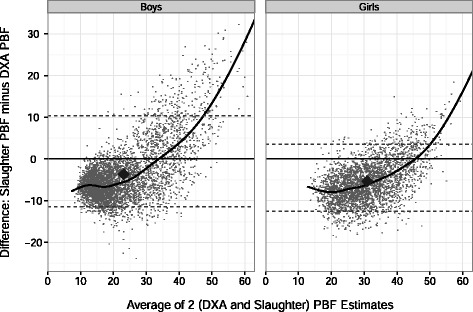
Bland-Altman plot for the agreement between the DXA and Slaughter estimates of percent body fat. Eachpoint represents an individual children and the black line is the smoothed (lowess) curve. The overall medians are shown by the large diamonds, and the dashed lines represent the 95% CI for the agreement between the 2 methods; if the estimates for the 2 methods were identical, all points would fall along the y=0 line. The PBF _Slaughter_ estimates appear to be biased, with PBF _Slaughter_ underestimating PBF _DXA_ among most children, but overestimating PBF _DXA_ among the heaviest children, particularly among boys

As seen in the Bland-Altman mean-difference plot (Fig. 1), the agreement between the Slaughter and DXA estimates of percent body fat varied substantially by the degree of body fatness. The largest underestimation of PBF_DXA_ occurred at low levels of body fatness. This underestimation decreased at higher levels of percent body fat, and at about 35 % (boys) and 45 % (girls) there was little difference between the 2 estimates. Among children (particularly boys) who had higher levels of percent body fat, PBF_Slaughter_ substantially overestimated PBF_DXA_. Additional analyses, stratified by sex and age group (<12 y, 12 to 13.9 y, and ≥14 y) indicated that within each sex-age group, the overestimation of PBF_DXA_ by PBF_Slaughter_ was most pronounce at low levels of body fatness, and the overestimation decreased as body fatness increased (data not shown).

We then examined differences between PBF_Slaughter_ and PBF_DXA_ within strata of the SF sum (Table 2). At relatively low (below the 33^rd^ percentile) levels of the SF sum (<17 mm, boys; <25 mm, girls), PBF_Slaughter_ underestimated PBF_DXA_ by 8 to 9 percentage points. The magnitude of this difference decreased at higher SF sum levels, and for children in the highest SF sum category, PBF_Slaughter_ overestimated PBF_DXA_ by about 10 percentage points among boys but only by 1.5 percentage points among girls.

**Table 2:** Levels of various characteristics within categories of the skinfold sum

Sex	SF Sum category (mm) ^a^	N	Age^b^	% Obese	% Extreme Obesity	SF sum (mm)	PBF_Slaughter_^b^	PBF_DXA_^b^	PBF Difference: Slaughter – DXA
Boys	<17	1548	13.3 ± 0.1	0	0	13.8 ± 0.1	11.2 ± 0.1	19.2 ± 0.1	−8.0
	17–27.4	1387	14.4 ± 0.1	2 ± 1	0	21.3 ± 0.2	17.8 ± 0.1	23.6 ± 0.2	−5.8
	27.5–49	1125	14.1 ± 1.5	38 ± 2	7 ± 1	36.6 ± 0.3	30.2 ± 0.2	32.2 ± 0.4	−2.0
	≥50	433	14.6 ± 0.2	92 ± 2	50 ± 4	60.1 ± 0.6	48.7 ± 0.6	38.9 ± 0.4	+9.7
Girls	<25	991	13.0 ± 0.1	0	0	18.9 ± 0.1	17.8 ± 0.1	26.6 ± 0.2	−8.8
	25–39	1090	14.6 ± 0.2	3 ± 1	0	31.9 ± 0.2	26.8 ± 0.1	33.3 ± 0.2	−6.4
	40–56	652	14.9 ± 0.2	38 ± 3	7 ± 2	47.1 ± 0.3	35.4 ± 0.2	39.3 ± 0.3	−3.9
	≥ 57	373	15.9 ± 0.3	87 ± 3	39 ± 4	67.1 ± 0.9	46.4 ± 0.5	44.8 ± 0.4	+1.5

^a^Cut-points for the SF sum categories approximately the 33rd, 67th, and 90th weighted percentiles within each sex

^b^Values are mean or prevalence ± SE within each SF sum category

**Fig. 2:**
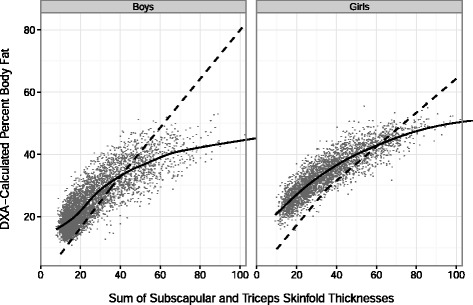
The relation of the SF sum to levels of PBF_DXA_ for each child (points), along with the predicted relationship of the SF sum to PBF _Slaughter_(dashed line) and PBF _DXA_(solid line, lowess). For boys with a SF sum < 35 mm, the intercept of the SF sum vs. PBF _Slaughter_ line varies by race and sexual maturation in the Slaughter equations,[26] and the illustrated line is for white, pubescent boys. Among pubertal (ages 12 to 13.9 y) boys who have a SF sum ≤ 35 mm, the estimated percent body fat is: -3.4 + 1.21*(SF sum) -0.008*(SF sum)2. For boys with a SF sum >35 mm, the equation is: 1.6 + 0.783*(SF sum) irrespective of pubertal stage

Figure 2 shows the relation of the SF sum to levels of PBF_DXA_ for each child (points), along with the relation of the SF sum to both PBF_Slaughter_ (dashed line) and PBF_DXA_ (solid line). As illustrated by the lowess curve (solid line), the association between SF sum and PBF_DXA_ was curvilinear, with the slope decreasing as the SF sum increased. In contrast, there were only small changes in the relation of SF sum to PBF_Slaughter_ (dashed line), with the slope decreasing from 0.84 to 0.78 at a SF sum of 35 mm among white boys and from 0.78 to 0.55 among girls. These differences in the slopes of the 2 lines resulted in PBF_Slaughter_ underestimating PBF_DXA_ among most children, but overestimating PBF_DXA_ among boys with a very high SF sum.

Table 3 shows mean levels of the CVD risk factors by sex and PBF_Slaughter_ category. As PBF_Slaughter_ increased, the prevalence of obesity varied from 0 to 58 % among boys and from 0 to 68 % among girls. Children in the highest PBF_Slaughter_ group also had adverse levels of PBF_DXA_ and the various CVD risk factors as compared to children in the lowest PBF_Slaughter_ group. With the exception of DBP, all risk factor differences between the lowest and highest PBF_Slaughter_ groups were statistically significant at the 0.01 level. Although the mean age of girls differed across the PBF_Slaughter_ categories, additional adjustment for age substantially influenced only levels of DBP, reducing the magnitude of the difference from 3 to 1 mm Hg among girls.

**Table 3:** Mean levels of obesity, body fatness, and CVD risk factors by categories of sex and percent body fat estimated from the Slaughter Equations

PBF_Slaughter_ Category	N_TC_^a^	Age (years)	Obese (%)	PBF_DXA_	Total Cholesterol (mg/dL	Triglycerides (mg/dL)^b^	Non-HDL Cholesterol (mg/L)	LDL Cholesterol (mg/dL)	HDL Cholesterol (mg/dL)	N_SBP_^a^	SBP (mm Hg)	DBP (mm Hg)
Boys												
<15 %	1703	14 ± 0.1^c^	0 ^c^	19 ± 0.1	156 ± 1	69 (66, 72)	103 ± 1	86 ± 1	53 ± 0.6	1825	107 ± 0.4	58 ± 0.5
15 - 24.9 %	1143	14 ± 0.2	4 ± 1	25 ± 0.2	161 ± 1	78 (73, 84)	112 ± 1	94 ± 2	49 ± 0.5	1208	108 ± 0.4	58 ± 0.7
≥ 25 %	1193	14 ± 0.2	58 ± 2*	35 ± 0.3*	172 ± 2*	110 (101,119)*	127 ± 1*	102 ± 2*	44 ± 0.6*	1287	113 ± 0.5*	59 ± 0.6
Girls												
<25 %	1122	13 ± 0.1	0	28 ± 0.2	162 ± 1	72 (67, 77)	107 ± 1	89 ± 2	56 ± 0.5	1255	102 ± 0.4	59 ± 0.5
25 - 34.9 %	960	15 ± 0.2	11 ± 1	35 ± 0.2	165 ± 1	79 (73, 86)	113 ± 1	92 ± 2	52 ± 0.5	1056	106 ± 0.6	60 ± 0.5
≥ 35 %	614	15 ± 0.2	68 ± 4*	43 ± 0.4*	170 ± 2*	84 (76, 92)*	122 ± 2*	99 ± 3*	47 ± 0.6*	679	110 ± 0.5*	62 ± 0.6

^a^ Ns in the column heading represent number of children with a non-missing value of that characteristic (total cholesterol or SBP). Ns for levels of TG and LDL-C, which required the child (age, 12–19 y) to be fasting, were about one third of the Ns for total cholesterol. The sample sizes for all risk factors are given in the Methods section

^b^ Geometric means are shown for TG levels, which were log-transformed

^c^ Values are mean or prevalence ± SE within each SF sum category

^*^ *P* < 0.01 for difference in CVD risk factor level between lowest and highest PBF_Slaughter_ categories based on linear or logistic regression models that controlled for age and 2-year cycle

Table 4 shows correlations between the levels of the various risk factors (columns) with levels of adjusted BMI, PBF_Slaughter_, and PBF_DXA_. (Regression models were used to adjust all characteristics for sex and age, and the values in the table represent the correlations between the residuals of these models.) With the exception of DBP, risk factor levels were significantly associated with the 3 body size measures. Furthermore, there was little difference in the relation of the 3 body size measures to levels of lipids and lipoproteins. For example, correlations with non-HDL cholesterol varied from *r* = 0.31 to 0.32 across the body size measure among boys and from *r* = 0.19 to 0.22 among girls.

**Table 4:** Correlations between the CVD risk factors and measures of body size, by sex

Sex	Characteristic	Total cholesterol	Triglycerides	LDL cholesterol	Non-HDL Cholesterol	HDL cholesterol	SBP	DBP
Boys	Adjusted BMI	0.20	0.39	0.24	0.31	−0.34	0.32	−0.01
	PBF_Slaughter_	0.21	0.40	0.25	0.32	−0.34	0.25*	0.02*
	PBF_DXA_	0.20	0.37	0.25	0.31	−0.33	0.27*	0.03*
Girls	Adjusted BMI	0.07	0.14	0.11	0.19	−0.31	0.32	−0.01
	PBF_Slaughter_	0.10	0.15	0.11	0.21	−0.29	0.21*	0.08*
	PBF_DXA_	0.11	0.15	0.15	0.22	−0.29	0.21*	0.05*

^a^ Levels of triglycerides were log transformed

* *P-*values assesses whether the correlation between the risk factor and adjusted BMI is equal to the correlation between the risk factor and either PBF_Slaughter_ or PBF_DXA_ . Among boys, for example, levels of SBP were more strongly associated with adjusted BMI (*r* = 0.32) than with PBF_DXA_ (*r* = 0.25). * *p* ≤ 0.01, H_**0**_: correlation of risk factor with adjusted BMI is equal to its correlation with PBF_Slaughter_ or PBF_DXA_

There were, however, differences in the magnitudes of the associations with blood pressure levels. SBP levels were more strongly associated with adjusted BMI than with levels of PBF_Slaughter_ or PBF_DXA_; among boys, for example, the 3 correlations were *r* = 0.32 (BMI), 0.25 (PBF_Slaughter_), and 0.27 (PBF_DXA_); *p* < 0.01 for both comparisons with adjusted BMI. Although levels of DBP were only weakly (*r* < 0.10) associated with any of the anthropometric variables, the associations were stronger for PBF_Slaughter_ and PBF_DXA_ than for adjusted BMI. Among girls, for example, the 3 correlations were *r* = -0.01 (BMI), *r* = 0.08 (PBF_Slaughter_) and *r* = 0.05 (PBF_DXA_).

There was also relatively little difference in the relation of the 3 body size measures to lipid and lipoprotein levels in analyses stratified by race-ethnicity. As seen in Table 5, as compared with PBF_Slaughter_ or PBF_DXA_, BMI was more strongly associated with levels of HDL cholesterol among white non-Hispanics, and with levels of both total and non-HDL cholesterol among Mexican-Americans. However, among black non-Hispanic children, BMI showed a weaker association with levels of LDL cholesterol than did PBF_Slaughter_.

**Table 5:** Correlations between the CVD risk factors and measures of body size, by race-ethnicity

Race-ethnicity	Characteristic	Total cholesterol	Triglycerides ^a^	LDL cholesterol	Non-HDL Cholesterol	HDL cholesterol	SBP	DBP
White non-Hispanics (*N* = 2026)	Adjusted BMI	0.14	0.31	0.17	0.26	−0.35	0.31	−0.03
	PBF_Slaughter_	0.17	0.33	0.17	0.27	−0.30*	0.21*	0.05*
	PBF_DXA_	0.17	0.25	0.16	0.26	−0.26*	0.22*	0.07*
Black non-Hispanics (*N* = 2433)	Adjusted BMI	0.11	0.32	0.20	0.24	−0.32	0.32	0.03
	PBF_Slaughter_	0.12	0.31	0.24*	0.26	−0.32	0.25*	0.07*
	PBF_DXA_	0.10	0.24	0.21	0.23	−0.31	0.21*	0.08*
Mexican-Americans (*N* = 2547)	Adjusted BMI	0.19	0.39	0.26	0.30	−0.32	0.35	0.00
	PBF_Slaughter_	0.19	0.37	0.25	0.30	−0.31	0.25*	0.04*
	PBF_DXA_	0.15*	0.32	0.19	0.25*	−0.30	0.24*	0.06*

^a^Levels of triglycerides were log transformed

* *P*-values assesses whether the correlation between the risk factor and adjusted BMI is equal to the correlation between the risk factor and either PBF_Slaughter_ or PBF_DXA_. Among white non-Hispanics, for example, levels of SBP were more strongly associated with adjusted BMI (*r* = 0.31) than with PBF_DXA_ (*r* = 0.22). * *p* ≤ 0.01, H_**0**_: correlation of risk factor with adjusted BMI is equal to its correlation with PBF_Slaughter_ or PBF_DXA_

## Discussion

It is sometimes asserted that body fatness is the true outcome of interest in obesity research and that BMI is an inaccurate surrogate. Although BMI is an inaccurate index of body fatness among normal-weight children [3], the results of several studies indicate that BMI is, in general, as strongly associated with adverse levels of various CVD risk factors as are more accurate assessments of body fatness [13–18]. In the current, cross-sectional study of 8- to 19-year-olds in the U.S., PBF_Slaughter_ estimates of body fatness were biased. PBF_Slaughter_ underestimated DXA-calculated percent body fat among relatively thin children, but the extent of underestimation decreased at higher levels of body fatness. Among the heaviest boys, PBF_Slaughter_ overestimated PBF_DXA_ by about 10 percentage points. Despite being less strongly associated with PBF_DXA_ than was PBF_Slaughter_, we found that adjusted levels of BMI were, in general, as strongly associated with levels of lipids and lipoproteins as was either PBF_Slaughter_ or PBF_DXA_. SBP levels, however, were more strongly associated with BMI, while the weaker associations (*r* < 0.10) with DBP levels were stronger for PBF_Slaughter_ and PBF_DXA_. These results are similar to our previous findings concerning among children in the Bogalusa Heart Study and the Pediatric Rosetta Body Composition Project [19].

In general, skinfold thicknesses (and estimates derived from them) are more strongly correlated with body fatness than is BMI, but some of the observed differences have been relatively small [3, 43]. Furthermore, the accuracy of skinfold thickness estimates of body fatness likely varies across skinfold sites and equations [21], in part due to differences in the distribution of body fatness [44]. For example, whereas various skinfold thicknesses and equations were stronger predictors of body fatness (determined from a 4-compartment model) than was BMI (R^2^s of ~0.85 vs. 0.67) [3], the multiple R^2^ for individual skinfolds varied from 0.76 (thigh) to 0.85 (biceps) [45].

It is possible that much of the discrepancy between PBF_Slaughter_ and PBF_DXA_ in the current study results from the relatively thin children and adolescents in the sample (*n* = 242) in which the Slaughter equations were developed [31]. Although BMI levels were not reported in this 1988 paper, these participants weighed less and had much thinner skinfolds than did those in the current analysis. For example, the mean SF sum among the 58 post-pubescent boys in the 1988 study was 18 mm (SD = 7) [31], whereas the mean SF sum among the 2572 14- to 19-year-old boys in the current study was 50 % larger (27 mm). It is unlikely that equations developed among relatively thin children can accurately estimate the body fatness of the much heavier children and adolescents in the current U.S. population.

In agreement with our results among the heaviest children, a previous analysis of data from the Pediatric Rosetta Body Composition Project obtained using Lunar models DPX and DPX-L [19] also found that the Slaughter skinfold thickness equations overestimate DXA-calculated percent body fat among heavy children. As shown in Fig. 2, this overestimation likely results from the functional form of the Slaughter equations. Although the Slaughter equations include a squared term for the SF sum [31], this term has very little influence on the estimated values. Furthermore, at SF sum values > 35 mm, the Slaughter equations are linear, with each 1 mm increase in the SF sum associated with a 0.783 (boys) or 0.546 (girls) increase in the estimate of percent body fat. As shown in Fig. 2, there is a nearly linear relationship between the SF sum and PBF_Slaughter_ throughout the entire range of SF sum values, while the relation of the SF sum to PBF_DXA_ is strongly curvilinear.

In general, the magnitudes of the associations with CVD risk factor levels that we observed agree fairly well with previous reports, including an analysis of NHANES 1999-2004 data that examined the relation of PBF_DXA_ to levels of lipids and lipoproteins [46]. Many investigators have found levels of various risk factors to be related similarly to levels of BMI and to estimates of body fatness calculated from skinfold thicknesses [17, 19], air-displacement plethysmography [13] and DXA [14–16]. This similarity may arise because the associations are largely influenced by risk factor levels among obese children, among whom BMI is a relatively good indicator of fatness [3], or because of the errors in measurement associated with skinfold thicknesses [5]. We did, however, observe some consistent differences in the associations with blood pressure, with BMI showing the strongest (*p* < 0.01) association with SBP but the weakest association with DBP.

There are additional limitations of the current, cross-sectional analyses that should be considered. Although the errors in the measurement of skinfold thicknesses are well known [5]. DXA estimates of the body fatness of an individual can also differ substantially from those obtained with the 4-compartment model and neutron activation [47]. It is also possible that DXA underestimates the body fatness of leaner persons and overestimates the fatness of obese persons [48], but if this occurred in the current study, the PBF_Slaughter_ overestimation of the body fatness of obese children may be even greater than what we observed. Although errors may have also been introduced by our use of age as a surrogate for pubertal maturation, we observed the largest discrepancies between PBF_DXA_ and PBF_Slaughter_ among boys with thick skinfolds; among these boys, PBF_Slaughter_ is based on only the SF sum [31]. It should also be realized that because BMI performs better as an indicator of body fatness among children who have relatively high levels of percent body fat than among thinner children [4, 20, 45], our results may not apply to populations in which the prevalence of obesity is relatively low.

## Conclusion

Our results indicate that the Slaughter skinfold thickness equations of percent body fat are biased, with PBF_Slaughter_ overestimating the body fatness of obese children, particularly obese boys. Furthermore, with the exception of very weak associations with DBP levels, adjusted (for sex and age) BMI values are as strongly associated with levels of various CVD risk factors as is PBF_Slaughter_. Our results do not support the possibility that the assessment of CVD risk among children and adolescents could be improved through the measurement of skinfold thicknesses or the use of DXA-calculated percent body fat rather than BMI.

## Abbreviations

BMI: Body mass index
CDC: Centers for disease control and prevention
DXA: Dual energy x-ray absorptiometry
NHANES: National health and nutrition examination survey
PBF_DXA_: Percent body fat estimated by dual energy X-ray absorptiometry
PBF_Slaughter_: Percent body fat estimated by the Slaughter skinfold thickness equations
